# A Novel Clinical Feature in NOG Gene Mutation-Associated Syndrome

**DOI:** 10.3390/audiolres15050130

**Published:** 2025-10-04

**Authors:** Matea Zrno, Tena Simunjak, Filip Bacan, Maja Lakus Ivancek, Jakov Ajduk

**Affiliations:** 1 Ministry of Defense of the Republic of Croatia, 10000 Zagreb, Croatia; matea.zrno@morh.hr; 2 Department of Otorhinolaryngology, Head and Neck Surgery, University Hospital Sveti Duh, 10000 Zagreb, Croatia; tsimunjak@kbsd.hr; 3 School of Medicine, University of Zagreb, 10000 Zagreb, Croatia; filip.bacan@kbcsm.hr; 4 SUVAG, 10000 Zagreb, Croatia; mlakus@suvag.hr; 5 Department of Otolaryngology and Head and Neck Surgery, University Hospital Center Sestre Milosrdnice, 10000 Zagreb, Croatia

**Keywords:** NOG gene, Teunissen-Cremers, mixed hearing loss, syndrome, stapes ankylosis

## Abstract

**Introduction:** Noggin encoding (NOG) gene plays a critical role in early embryogenesis and development of bones, joints, cartilage, eyes, and neural tissue. The NOG gene encodes the noggin protein. Noggin is the only secreted inhibitor of bone morphogenetic protein (BMP) that is associated with abnormal phenotypes in humans. The most commonly observed manifestations of NOG gene mutations include bilateral conductive hearing loss, proximal symphalangism, broad thumbs, hyperopia, and a distinct facial appearance. This genetic disorder was first reported in 1990 by Teunissen and Cremers. Since then, various phenotypic presentations of NOG mutation have been reported, leading to the introduction of the term NOG-related symphalangism spectrum disorder (NOG-SSD). Case report: In this report, we describe a family (mother and daughter) with bilateral mixed hearing loss. Both patients had hyperopia, distinct facial appearance with hemicylindrical nose, broad thumbs, and syndactyly of the second and third toes. Genetic testing confirmed a NOG gene mutation. Bilateral stapedotomy was successfully performed, resulting in significant hearing improvement. However, due to sensorineural component of hearing loss, complete hearing recovery was only achieved with the use of hearing aids. **Discussion:** The etiology of the sensorineural component of hearing loss in NOG-SSD remains unclear. In animal models, the NOG gene is essential for inner ear development, while in humans, only middle ear malformations have been reported. The phenotypic variability observed in individuals with NOG mutations is very wide, suggesting that the sensorineural component of hearing loss could represent one of the possible manifestations. **Conclusions:** Conductive hearing loss is the primary manifestation of the NOG-SSD, and all previously reported cases of NOG gene mutations have presented exclusively with conductive hearing loss. It is possible that additional genetic factors, not necessarily directly related to the NOG gene but present in this family, contribute to the development of the sensorineural component of hearing loss, although thorough genetic testing did not reveal any additional mutation. This is, to our knowledge, the first report of mixed hearing loss associated with a NOG mutation confirmed preoperatively. Further studies are needed to determine whether the sensorineural component represents a primary manifestation or arises from secondary mechanisms.

## 1. Introduction

Noggin encoding (NOG) gene has a critical role in early embryogenesis and the development of bones, joints, neural tissue, skeletal muscles, cartilage, hair follicles, and craniofacial structures such as telencephalon and eyes [1,2].

A mutation of the NOG gene leads to absence of the noggin protein, a polypeptide crucial for regulating multiple signaling pathways during human development, particularly in cartilage and bone formation [1]. Noggin binds to and inactivates bone morphogenetic protein (BMP), and this interaction is essential for normal embryogenesis [1]. BMP regulates the differentiation of mesenchymal cells, activation of osteoblasts, and induction of apoptosis at joint sites. In the absence of noggin, BMP signaling remains unregulated, leading to chondrocyte hyperplasia instead of apoptosis in the joint [1]. Noggin is the only secreted BMP inhibitor associated with abnormal phenotypes in humans [1].

In animal models, the NOG gene is essential for nervous system development, influencing neuronal differentiation and survival [3]. It regulates BMP signaling, which is crucial for sensory neuron function [3,4].

Mutations in the *NOG* gene result in various autosomal dominant syndromes characterized by a variety of skeletal dysplasia. Several clinical syndromes associated with NOG mutation have been described, including proximal symphalangism (defined by abnormal fusion of the proximal interphalangeal joints of the hands and feet), multiple synostoses syndrome, tarsal–carpal coalition syndrome, and stapes ankylosis with broad thumbs and toes (Teunissen Chremers syndrome) [5,6,7]. Additional clinical features involving facial structure, hearing loss, and vision have been reported: broad, hemicylindrical nose, asymmetric mouth, hyperopia, strabismus, and conductive hearing loss [7,8]. The most common visual impairments are hyperopia and strabismus. Due to the wide phenotypic variability, a unifying term was introduced: NOG-related symphalangism spectrum disorder (NOG-SSD) [9]. This term encompasses multiple disorders with the *NOG* gene as the common molecular etiology, highlighting the variable spectrum of associated findings.

Mutations of the *NOG* gene are recorded in a locus-specific database (https://NOG.lovd.nl, accessed on 21 July 2025). Public databases such as dbSNP, 1000 Genome Browser, HGVD, ESP6500, and ExAC are used to evaluate variant allele frequency [10].

The incidence of congenital ossicular chain anomalies causing conductive hearing impairment ranges between 0.5% and 1.2%, with most anomalies being non-hereditary [11].

Middle ear malformations have been classified into four main groups: isolated stapes ankylosis, stapes ankylosis associated with other ossicular malformations, deformity of the ossicular chain with mobile stapes footplate, and severe aplasia or dysplasia of oval or round windows [11,12,13]. The most common middle ear finding in patients with NOG gene mutations is stapes fixation (ankylosis). Histological reports on resected stapes demonstrated an abnormal bony fusion of the stapes footplate with thickened bone in the oval window niche and calcification of the annular ligament [12,13]. Altered binding of mutant noggin may lead to hyperactivation of BMP signaling, ultimately causing stapes ankylosis. Animal model studies have shown that the NOG gene is responsible for stapes and cochlear duct formation in the inner ear as well as for hair cell development in mouse cochlear explants [14,15]. However, the role of BMP and noggin in development of human inner ear remains unclear. Audiological evaluation typically demonstrates conductive hearing loss, absent stapedial reflexes, and absent otoacoustic emissions. The severity of hearing loss ranges from 20 to 80 dB, with most affected individuals experiencing moderate hearing loss. Hearing loss due to the NOG gene mutation is often misdiagnosed as otosclerosis.

High-resolution computer tomography (HRCT) and magnetic resonance imaging (MRI) of the temporal bones are frequently normal in patients with NOG-SSD. Occasionally, HRCT may reveal oval window narrowing and stapes deformity, though imaging is more useful for excluding other causes of conductive hearing loss. Sensorineural component hearing loss has not previously been associated with NOG gene mutations. Progressive sensorineural hearing loss has only been described in few patients with NOG gene mutations after stapes surgery and this was attributed to perilymph fistula [16].

We present a case of mother and daughter with mixed hearing loss, stapes ankylosis, specific facial characteristic, proximal symphalangism, and hyperopia.

Given their similar physical features and bilateral hearing loss, genetic testing was performed, confirming a NOG gene mutation (deletion of C nucleotide on c.291 position).

While previous reports have only described conductive hearing loss in NOG gene mutations, this family presented with mixed hearing loss. This may represent a novel manifestation of NOG gene mutations contributing to the sensorineural component of hearing loss.

## 2. Case Report

**Figure 1:**
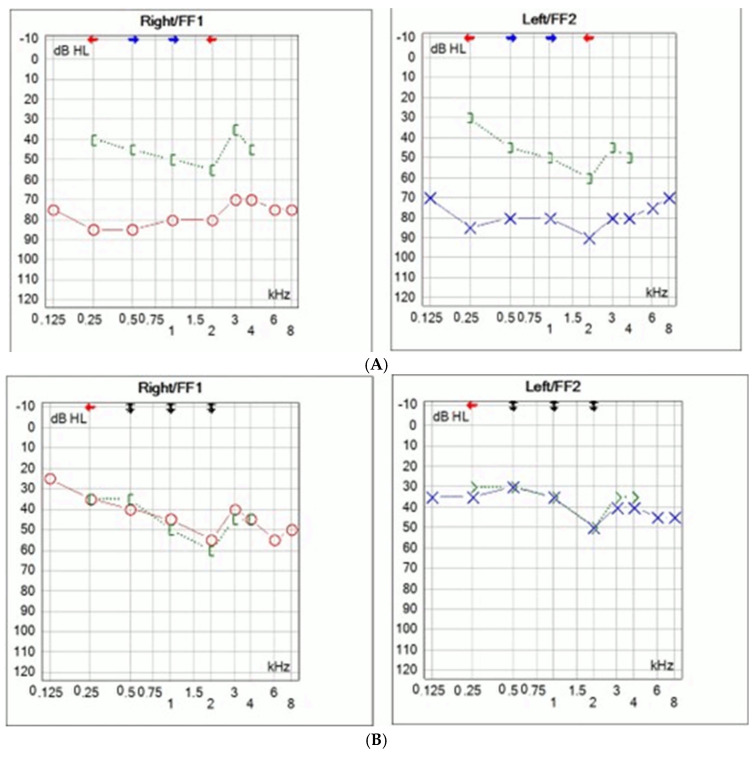
Preoperative pure tone audiometry of the mother (age 25) showing bilateral mixed hearing loss (**A**). Postoperative pure tone audiometry demonstrating significant improvement in hearing threshold following bilateral stapedotomy, with closure of the air–bone gap residual sensorineural hearing loss (**B**).

A seven-year-old girl with bilateral hearing loss was referred to our clinic. She had been using bilateral hearing aids since early childhood, but the hearing progressively deteriorated. Her mother also had bilateral mixed hearing loss from childhood and underwent surgery at the age of 25, ten years prior (Figure 1). Both the girl and her mother displayed mild dysmorphic facial characteristics, including hemicylindrical nose, hyperopia, strabismus, wide thumbs and toes, and partial syndactyly of second and third toes. The partial syndactyly of second and third toe caused no functional impairment, so no treatment was required. No other joint or bone abnormalities were detected. The late grandmother had similar facial characteristics and hearing difficulties, although no audiological testing was performed. The mother had undergone bilateral stapedotomy ten years earlier (Figure 1A).

Intraoperatively, bilateral stapes ankylosis was found, without other ossicular malformations. Stapedotomy was performed using a fluoroplastic piston prosthesis (Medtronic, Boston, MA, USA). No perylimphatic gusher was noticed. Significant improvement of hearing was achieved with complete closure of air–bone gap (Figure 1B).

Hearing remained stable for the following ten years; no additional deterioration of hearing loss was detected. At that time, no suspicion of genetic mutation was raised. However, due to the sensorineural component of hearing loss, full hearing recovery was only achieved with hearing aids. Hearing loss in daughter was discovered at the age of two. Given the family history and phenotypic features, genetic testing was performed. GJB2 variants were analyzed with the multiplex ligation-dependent probe amplification method and Sanger sequencing of the coding region of the GJB2 gene (MRC Holland, Amsterdam, The Netherlands). For clinical exome sequencing (CES) analysis, the DNA library was generated with enrichment oligonucleotides using Illumina DNA Prep with Enrichment, focusing on the exons of 4813 disease-associated genes (TruSight One Panel, Illumina Inc., San Diego, CA, USA).

Genetic analysis revealed a heterozygous deletion of C nucleotide at c.291 position in the NOG gene (NOG: *c.(291delc);(291=)*, *p.(Ala98ArgfsTer26)* in both mother and daughter. No other mutations were detected. The father and son tested negative.

**Figure 2:**
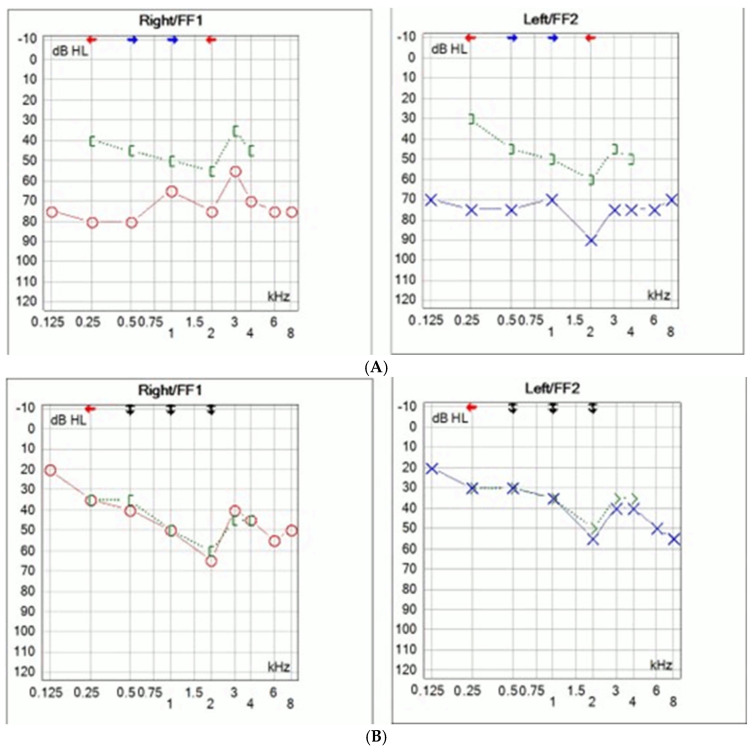
Preoperative pure-tone audiometry of the daughter (age 7) showing bilateral mixed hearing loss (**A**). Postoperative pure tone audiometry demonstrating significant improvement in hearing threshold following bilateral stapedotomy, with closure of the air–bone gap, and residual sensorineural hearing loss (**B**).

Comprehensive audiological testing for the daughter has been conducted. Pure tone audiometry was performed (Interacoustic AS, Middelfart, Denmark) and bilateral mixed hearing loss was determined (Figure 2A). The left ear hearing thresholds ranged between 70 and 90 dB with an air–bone gap of 30–40 dB, while the right ear hearing threshold ranged between 50 and 80 dB, with an air–bone gap of 30–40 dB (Figure 2A).

Stapedial reflexes and otoacoustic emission were absent. HRCT of the temporal bones revealed no malformation. MRI of the brain and temporal bones was normal.

Due to inadequate hearing with hearing aids surgical treatment was proposed. Bilateral middle ear exploration was performed through transmeatal approach. Intraoperative findings revealed bilateral stapes ankylosis without other ossicular malformations. Stapedotomies were performed with manual perforator. Fluoroplastic piston stapes prosthesis was used (Grace Medical, Inc., Memphis, TN, USA). Since simultaneous bilateral surgeries are not recommended, the surgeries were staged six months apart. No perylimphatic gusher was observed. Post operative audiometry showed significant improvement in hearing thresholds with complete closure of the air–bone gap (Figure 2B). Bone conduction thresholds remained unchanged. Hearing remained stable during the one year follow-up.

## 3. Discussion

Most patients with NOG mutation were initially diagnosed with conductive hearing loss due to otosclerosis. However, studies have shown that NOG gene mutations are not present in patients with otosclerosis, suggesting that these mutations are restricted only to individuals with skeletal abnormalities [11]. Therefore, molecular genetic testing is crucial for differentiating syndromic stapes ankylosis from otosclerosis. Even subtle skeletal anomalies may point to NOG-associated disorders [12].

**Figure 3:**
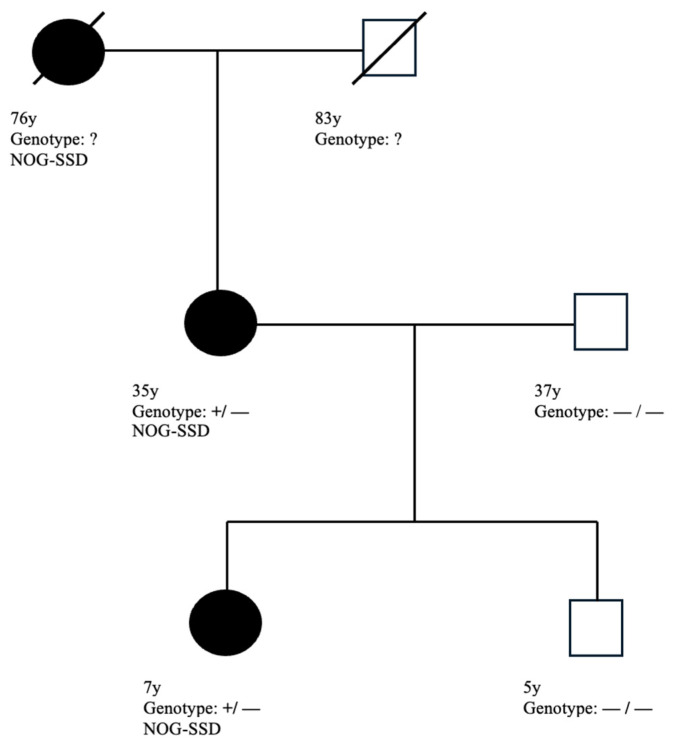
Pedigree of the family with ages and genotypes. Squares denote males, circles denote females. Filled symbols indicate affected individuals with clinical manifestations consistent with NOG-SSD. Line across a symbol indicates deceased individuals. Ages are shown below each symbol (for deceased individuals, the age at death is indicated). Genotypes are given as follows: +/− = heterozygous for *c.291delC (p.Ala98ArgfsTer26)* variant; −/− = wild type; ? = not tested.

Diagnosing hearing loss due to genetic mutation can be challenging. NOG gene mutations follow an autosomal-dominant inheritance pattern. Teunissen and Cremers first reported this syndrome in 1990 in a family with five males across three generations, all of whom all had stapes ankylosis, hyperopia, broad thumbs, broad first toes, and syndactyly [6,7]. It was named the Teunissen–Cremers syndrome. Subsequent genetic studies demonstrated that NOG gene mutations cause several bone disorders such as stapes ankylosis with broad thumbs and toes (SABTT), proximal symphalangism (SYM1), multiple synostoses syndrome 1 (SYNS1), tarsal–carpal coalition syndrome (TCC), and brachydactyly type B2 (BDB2) [9,17]. These phenotypes are now collectively referred to as NOG-related syndromes [9]. To date, 45 variation of NOG gene mutations in human have been reported, and the term NOG-SSD has been introduced to describe the spectrum of these disorders [18,19]. Mild facial dysmorphism such as observed in our patients, can easily be overlooked, particularly when clinicians focus primarily on hearing loss. Other subtle physical features, including myopia and toe syndactyly, can also be very easily overlooked. In this report, we describe three affected female patients across three generations. Although genetic testing was not performed in the grandmother, her clinical features strongly support familial inheritance. Genetic testing was only considered when the daughter presented with similar audiological and other clinical characteristics as her mother. The pedigree of the family is shown in Figure 3.

Previous research has not demonstrated gender predisposition for NOG gene mutations, which affects both males and females equally [12].

Stapes surgery is a successful treatment for the patients with conductive hearing loss caused by stapes ankylosis [12]. Many studies have confirmed that the stapedotomy leads to significant hearing improvement [12,18]. However, postoperative long-term follow up has shown cases of bony reclosure of the oval window and the dislocation of the piston prosthesis [19]. Excessive perilymph leakage during surgery has also been reported in patients with NOG gene mutations [12]. Postoperative progressive sensorineural hearing loss has been described in only few cases, and it was attributed to perilymph fistula [12,16]. Other middle ear ossicle malformations have been reported in patients with NOG gene mutations, including fixation of the short process of incus, malleoincudal joint fixation, and elongation of the long process of incus [9,12]. Ear surgery remains the treatment of choice for congenital ossicular chain anomalies. Hearing aids are indicated in patients who cannot undergo surgery or, as in our cases, as an adjunct therapy. Implantable hearing aids are an option when ossiculoplasty or stapedotomy fail to provide sufficient improvement.

According to the public mutation database CLINVAR and the literature, the NOG gene mutation detected in this family (*c.291delC*, *p.Ala98ArgfsTer26*) has not been previously described [9,18,19]. The American College of Medical Genetics and Genomics (ACMG) recommended five categories for variant classification: pathogenic, likely pathogenic, uncertain significance, likely benign, and benign [20,21]. We therefore applied the ACMG guidelines to evaluate its pathogenicity [20,21]. The variant detected in our family (*c.291delC*, *p.Ala98ArgfsTer26*) was evaluated using the standardized framework [20,21]. The following criteria were considered: PVS1 (very strong): this is a frameshift variant predicted to result in a premature stop codon and subsequent nonsense-mediated decay, supporting pathogenicity; PM2 (moderate): the variant is absent from large population databases, including gnomAD, ExAC, 1000 Genomes, and ESP6500; and PP4 (supporting): the clinical presentation of the affected individuals (stapes ankylosis, broad thumbs and toes, hyperopia, and characteristic facial features) is highly specific and consistent with NOG-SSD. However, the classification cannot be upgraded to “likely pathogenic” or “pathogenic” due to the following limitations: the variant has not been previously reported in ClinVar, LOVD, or the published literature; functional studies confirming the pathogenic mechanism are lacking; and segregation data are limited to two affected individuals within a single family. Based on this evidence, the variant is classified as a Variant of Uncertain Significance (VUS), fulfilling the criteria for PVS1, PM2, and PP4, but remaining uncertain due to the absence of functional studies and insufficient segregation data.

Previous studies of NOG gene mutations described only conductive hearing loss due to stapes ankylosis, whereas in our family, mixed hearing loss was observed [22]. While patients with NOG gene mutations do have vision deficits such as hyperopia and strabismus, sensorineural hearing loss has not been previously described as part of the NOG-SSD [9,19].

In animal models, the NOG gene is required for the formation of the stapes, cochlear duct in the inner ear, nervous system development, and neuronal differentiation [3,4]. It regulates BMP signaling, which is crucial for sensory neuron function [3]. In humans, the possible role of the NOG gene in formation of inner ear structures and nervous system development is not completely known [9].

The phenotypic variability observed in individuals with NOG mutations, together with the role of noggin and BMP in development of mouse inner ear, suggests that NOG gene mutations contribute to the sensorineural component of hearing loss in humans.

Long-standing or severe conductive hearing loss, associated with stapes fixation in patients with otosclerosis can impact inner ear function and induce sensorineural hearing loss but in cases of NOG gene mutations, histopathology of temporal bone showed no evidence of inner ear pathology [23]. Since mixed hearing loss was established at a very early age in our patients, the sensorineural component cannot be explained by long-standing conductive hearing loss. Additional genetic factors, not necessarily directly related to the *NOG* gene, may contribute to the development of the sensorineural component of hearing loss, although thorough genetic testing did not reveal other mutations.

## 4. Conclusions

Conductive hearing loss is the primary manifestation of NOG-SSD, caused by abnormal bony fusion and stapes fixation. However, significant phenotypic variability has been observed in individuals with NOG mutations, and mixed hearing loss could represent one of the possible manifestations. This is, to our knowledge, the first report of mixed hearing loss associated with a NOG mutation confirmed preoperatively. Further studies are needed to determine whether the sensorineural component represents a primary manifestation or arises from secondary mechanisms. It is possible that there are more patients with mixed hearing loss and NOG gene mutations, but they are not recognized. Further research is required to clarify whether the sensorineural component of hearing loss observed in this family is a direct consequence of *NOG* gene mutations. We presented this report not only because of its rarity but also to raise awareness among clinicians of the importance of considering genetic causes of hearing loss, particularly in young patients, where delayed diagnosis and treatment may significantly affect speech and language development.

## Data Availability

Data are available upon request from the authors.
